# Saw Palmetto Extract Inhibits Metastasis and Antiangiogenesis through STAT3 Signal Pathway in Glioma Cell

**DOI:** 10.1155/2015/926946

**Published:** 2015-12-15

**Authors:** Hong Ding, Jinglian Shen, Yang Yang, Yuqin Che

**Affiliations:** ^1^Department of Nephrology, The Fourth Affiliated Hospital of China Medical University, Shenyang, China; ^2^Department of Emergency Medicine, The Fourth Affiliated Hospital of China Medical University, Shenyang, China; ^3^Department of Neurology, The Fourth Affiliated Hospital of China Medical University, Shenyang, China

## Abstract

Signal transducer and activator of transcription factor 3 (STAT3) plays an important role in the proliferation and angiogenesis in human glioma. Previous research indicated that saw palmetto extract markedly inhibited the proliferation of human glioma cells through STAT3 signal pathway. But its effect on tumor metastasis and antiangiogenesis is not clear. This study is to further clear the impact of saw palmetto extract on glioma cell metastasis, antiangiogenesis, and its mechanism. TUNEL assay indicated that the apoptotic cells in the saw palmetto treated group are higher than that in the control group (*p* < 0.05). The apoptosis related protein is detected and the results revealed that saw palmetto extract inhibits the proliferation of human glioma. Meanwhile pSTAT3 is lower in the experimental group and CD34 is also inhibited in the saw palmetto treated group. This means that saw palmetto extract could inhibit the angiogenesis in glioma. We found that saw palmetto extract was an important phytotherapeutic drug against the human glioma through STAT3 signal pathway. Saw palmetto extract may be useful as an adjunctive therapeutic agent for treatment of individuals with glioma and other types of cancer in which STAT3 signaling is activated.

## 1. Introduction

Human gliomas which originate from neural stromal cells are the most common and malignant brain tumor in human [1]. Human gliomas account for 35.26–60.96% of central nervous system tumors (average, 44.69%). The incidence rate in adults is about 6/100,000 and the five-year survival rate is between 20 and 30% [2]. Due to the tumor's infiltrating growth and no evident boundary with the normal brain tissue, it is difficult to be removed completely via surgery. In addition, gliomas are not susceptible to radiotherapy or chemotherapy, which makes it the worst prognoses in systemic tumors [3]. Therefore, it is urgently needed to identify the critical carcinogenic pathways and discover novel treatment strategies for glioma.

The growth and proliferation of glioma cells are highly dependent on angiogenesis [4]. The glioma has a strong ability in promoting the formation of angiogenesis and microvascular network to provide the tumor with nutrients for the sake of further invasion and metastasis. So the treatment of antiangiogenesis therapy has become an important method in glioma.

Signal transducer and activator of transcription factor 3 (STAT3) with tyrosine phosphorylation in signal pathway exists in the cell holder, regulating the expression of a variety of functional protein, cell proliferation, and apoptosis. The present study has proven that there is a close relationship between STAT3 and cell adhesion molecules, extracellular matrix degrading enzymes, tumor angiogenesis, metastasis through MMP, VEGF, and other related gene interactions [5–7].

Saw palmetto (SR) is a kind of palm plant in Southeast American. In in vitro experiments people found that saw palmetto extract can effectively inhibit the proliferation with dose dependent relationship in human breast cancer cells [8]. In previous research, the effect of saw palmetto extract on human glioma U87 and U251 cells was investigated in vivo. The results revealed that saw palmetto extract markedly inhibited the proliferation of human glioma cells. The underlying mechanism may be associated with the inhibition of signal transducer and activator of transcription 3 phosphorylation [9].

As mentioned, saw palmetto extract regulates the proliferation of tumor cells by inhibiting the STAT3 signaling pathway, but its effect on tumor metastasis and antiangiogenesis is not clear. The purpose of this study is through researching the effect of saw palmetto extract on glioma cell migration related protein and marker of angiogenesis to further clear the impact of saw palmetto extract on glioma cell metastasis, antiangiogenesis, and its mechanism.

## 2. Materials and Methods

### 2.1. Animals and Cell Lines

30 healthy SD male rats were purchased from China Medical University Department of Experimental Animal, which weighed 250–300 g. The rats were randomly divided into three groups: control group, low dose SR group, and high dose SR group. Human glioma cell lines U87 and U251 were purchased from Beijing Dingguochangsheng Biotech Co., Ltd. (Beijing, China).

### 2.2. Reagents and Drug

Saw palmetto extract was purchased from Yongyuan Bio-technology, Co., Ltd. (Xi'an, China). TUNEL kit, rabbit anti-B-cell lymphoma-2 (Bcl-2), anti-CD34, anti-MMP-2, anti-PARP, and anti-pSTAT3 antibodies were purchased from the Beyotime Institute of Biotechnology (Shanghai, China).

### 2.3. Cell Culture

Human glioma cell lines, U87 and U251, were grown in a 25 cm^2^ cell culture bottle containing Dulbecco's modified Eagle's medium (DMEM), supplemented with 10% fetal bovine serum, 100 IU/mL penicillin, and 100 *μ*g/mL streptomycin. The cell was cultured at 37°C and 5% carbon dioxide. The medium was replaced every two days.

### 2.4. Cell Count

Blood cell counting plate and cover glass were cleaned with 95% alcohol. Take 1 *μ*L of cell suspension on the blood cell counting chamber to count the cell concentration. The cell number was counted with four angles in the grid on the plate. The procedure was performed in triplicate. The cell number was calculated as follows: Cell number (/mL) = (total cell number of the four angles/4)  *∗*  10^4^ *∗*  dilution.

### 2.5. Establishment of the Rat Glioma Model

The vigorous growing cell was collected; the cell concentration was adjusted to 1*∗*10^7^/mL. The cell suspension was subcutaneously injected into rat dorsal to establish glioma model. The control group was injected with equal amount of saline at the same site. SR low dose group and high dose SR group rats received administration of 50 mg/kg and 300 mg/kg saw palmetto extract every day through gastrointestinal tract for 4 weeks; meanwhile the control group rats were given the same amount of drinking water every day. After the last administration, the rats were sacrificed 24 hours later and the glioma specimens were collected.

### 2.6. TUNEL Assay

TUNEL assay was utilized to detect the apoptosis in glioma tissues. The tissues were fixed with 10% formalin, were dehydrated, were transparent, and were embedded in paraffin; then the specimens were cut into 5–8 *μ*m thick slices. Then the glioma sections were deparaffinized, rehydrated, and pretreated with 20 *μ*g/mL proteinase K. The endogenous peroxidase was blocked by 3% hydrogen peroxide. The specimens were subsequently incubated with terminal deoxynucleotidyl transferase (TdT) and reacted at 37°C for 1 hour, followed by antidigoxigenin antibody at 37°C for 30 min. After washing, slides were incubated with streptavidin-biotin-peroxidase for 20 min, stained with 3,3′-diaminobenzidine tetrahydrochloride, and counterstained with hematoxylin. Finally, the sections were dehydrated, coverslipped, and observed. Five sights of each section were randomly selected under microscope. The number of TUNEL-positive glioma nuclei and the total glioma nuclei in each sight were counted. The ratio of apoptotic cell was calculated by dividing the number of TUNEL-positive glioma nuclei by the number of total glioma nuclei.

### 2.7. The Expression of STAT3, MMP-2, CD34, and Bcl-2 Was Detected by Immunohistochemistry

All specimens were promptly fixed in 10% buffered formalin and embedded in paraffin. 4 *μ*m section was cut and conventionally dewaxed to water. The section was incubated in 3% of H_2_O_2_ PBS at room temperature for 10 min and then washed for five minutes with distilled water three times. The sections were immersed in 0.01 mol/L citric acid buffer (pH 6) boiled for 15 minutes with microwave. After cooling the specimen was washed 2 times with PBS. 5% of BSA sealing liquid was dropped on the specimen and left for reaction for 20 min at room temperature. After that the excess liquid was rejected with no washing. Diluted first antibody (rabbit anti-rat pSTAT3 (1 : 300), rabbit anti-rat Bcl-2 (1 : 300), rabbit anti-rat CD34 (1 : 300), rabbit anti-rat MMP-2 (1 : 300), and rabbit anti-rat PARP (1 : 300)) were added and reacted at 4°C in the moisturizing box overnight. The specimen was washed with PBS three times for 2 min the next day. Following primary antibody incubations, sections were incubated with biotin-conjugated secondary antibodies: goat anti-rabbit IgG (Beyotime Institute of Biotechnology, Shanghai, China) at a temperature of 37°C for 30 min in the moisturizing box. Following that the section was washed 3 times for 2 min with PBS. SABC reagents were added on the section and reacted for 20 min at 37°C in the moisturizing box. Then the section was washed 4 times for 5 min with PBS. Visualization of the immune complex involved the DAB kit according to the protocol. At last, the section was washed with distilled water and counterstained with hematoxylin staining.

### 2.8. Image Analysis

pSTAT3, Bcl-2, CD34, MMP-2, and PARP positive nuclei were brown or dark brown. The immunoreactive positive cells were determined by the average optical density value of ImageJ image analysis software.

### 2.9. Statistical Analysis

All the experimental data were processed by SPSS 19 statistical analysis software; the values are expressed in mean ± standard deviation. Comparison between groups was analyzed by one-way ANOVA, with statistically significant difference in *p* less than 0.05.

## 3. Results

### 3.1. The Apoptosis of Glioma Tissue Induced by Saw Palmetto Extract

**Figure 1:**
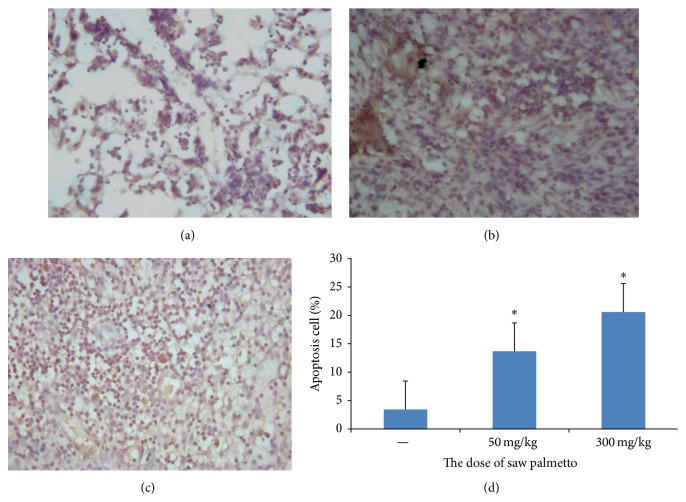
The apoptosis of glioma cell induced by saw palmetto through TUNEL assay. (a) is the control group, (b) is the low dose group in which the rats received administration of 50 mg/kg saw palmetto extract every day for 4 weeks, and (c) is the high dose group in which the rats received administration of 300 mg/kg saw palmetto extract every day for 4 weeks. (d) is the histogram to evaluate the apoptosis cell induced by saw palmetto of above three groups. *∗* means *p* < 0.05.

The apoptosis of glioma cell induced by saw palmetto was detected by TUNEL assay. As shown in Figure 1, the apoptosis ratio in the control group is 3.45 ± 0.48%, in the low dose group is 13.67 ± 0.34%, and in the high dose group is 20.58 ± 1.53%. The apoptotic cells in the saw palmetto treated group are higher than that in the control group (*p* < 0.05). In addition, we can conclude that the apoptosis ratio is higher in the high dose group than that in the low dose group (*p* < 0.05).

### 3.2. The Expression of pSTAT3 in the Glioma Tissue Was Measured by Immunohistochemistry Assay

**Figure 2:**
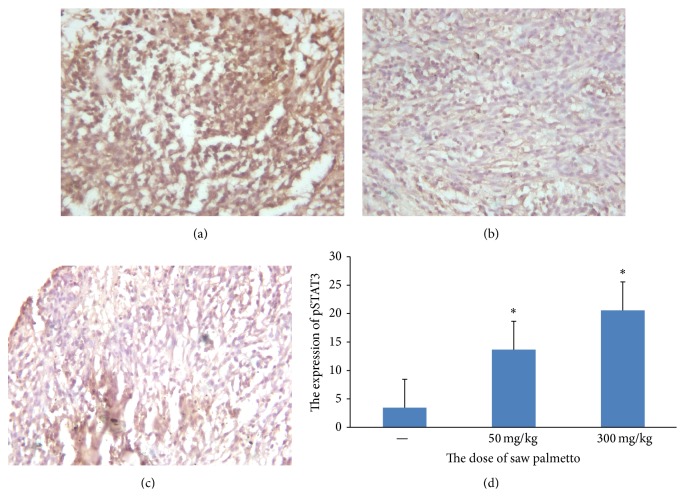
The expression of pSTAT3 in the glioma tissue. (a) is the control group, (b) is the low dose group in which the rats received administration of 50 mg/kg saw palmetto extract every day for 4 weeks, and (c) is the high dose group in which the rats received administration of 300 mg/kg saw palmetto extract every day for 4 weeks. (d) is the histogram to evaluate the expression of pSTAT3 in the three groups. *∗* means *p* < 0.05.

Previous study suggested that saw palmetto induced growth arrest and apoptosis of prostate cancer cells by the inhibition of STAT3 signal pathway. We adopted immunohistochemistry assay to measure the expression of pSTAT3 in glioma tissue. The results revealed that the optical density value is 0.295 ± 0.007 in the control group, 0.237 ± 0.005 in the low dose group, and 0.122 ± 0.008 in the high dose group (see Figure 2). The expression of pSTAT3 is lower in the experimental group than that in the control group (*p* < 0.05). At the same time we can see that the expression of pSTAT3 is negative with the concentration of saw palmetto.

### 3.3. The Expression of MMP-2 in the Glioma Tissue Was Measured by Immunohistochemistry Assay

**Figure 3:**
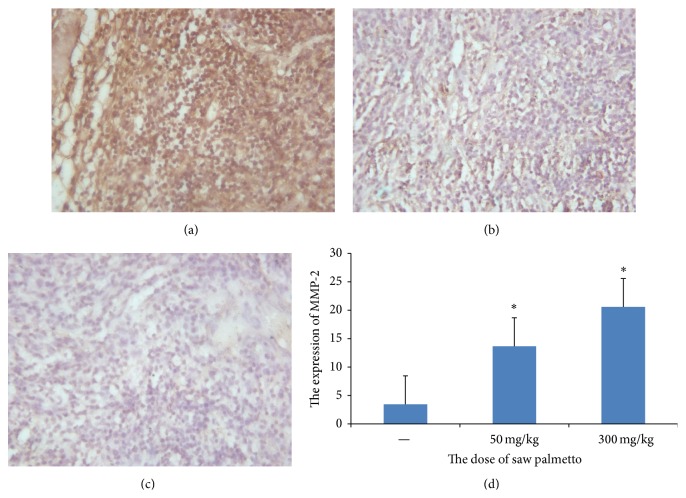
The expression of MMP-2 in the glioma tissue. (a) is the control group, (b) is the low dose group in which the rats received administration of 50 mg/kg saw palmetto extract every day for 4 weeks, and (c) is the high dose group in which the rats received administration of 300 mg/kg saw palmetto extract every day for 4 weeks. (d) is the histogram to evaluate the expression of MMP-2 in the three groups. *∗* means *p* < 0.05.

The previous study certified that MMP-2 is associated with the metastasis of tumor. We detected the expression of MMP-2 to evaluate the effect of saw palmetto on the metastasis of glioma. We adopted immunohistochemistry assay to measure the expression of MMP-2 in glioma tissue. The results revealed that the optical density value is 0.299 ± 0.009 in the control group, 0.222 ± 0.014 in the low dose group, and 0.122 ± 0.009 in the high dose group (see Figure 3). The expression of MMP-2 is lower in the experimental group than that in the control group (*p* < 0.05).

### 3.4. The Expression of CD34 in the Glioma Tissue Was Measured by Immunohistochemistry Assay

**Figure 4:**
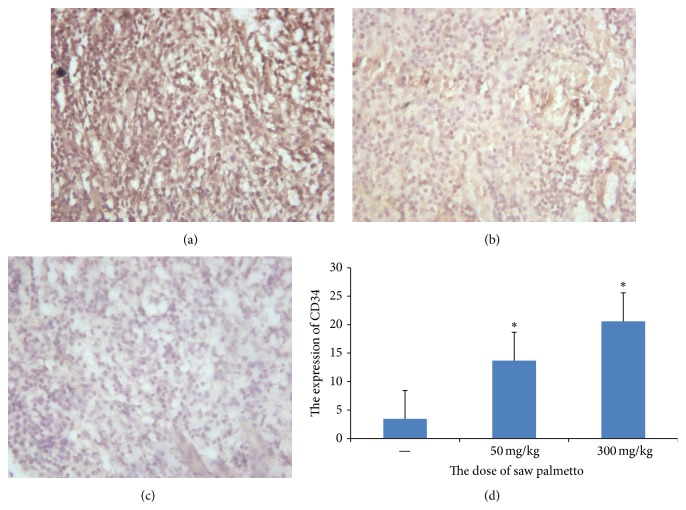
The expression of CD34 in the glioma tissue. (a) is the control group, (b) is the low dose group in which the rats received administration of 50 mg/kg saw palmetto extract every day for 4 weeks, and (c) is the high dose group in which the rats received administration of 300 mg/kg saw palmetto extract every day for 4 weeks. (d) is the histogram to evaluate the expression of CD34 in the three groups. *∗* means *p* < 0.05.

Angiogenesis is very important in the tumor cell proliferation and metastasis. As known, CD34 is a good marker to reflect the density of blood vessels in the tumor. In order to study the effect of saw palmetto on angiogenesis, we measure the CD34 by immunohistochemistry method. The results revealed that the optical density value is 0.288 ± 0.014 in the control group, 0.224 ± 0.011 in the low dose group, and 0.120 ± 0.115 in the high dose group (*p* < 0.05) (see Figure 4). The results means that the vascular density is lower in the experimental group which indicated that saw palmetto could inhibit the angiogenesis.

### 3.5. Effect of Saw Palmetto on Expression of Apoptotic Related Protein in Glioma Cells

**Figure 5:**
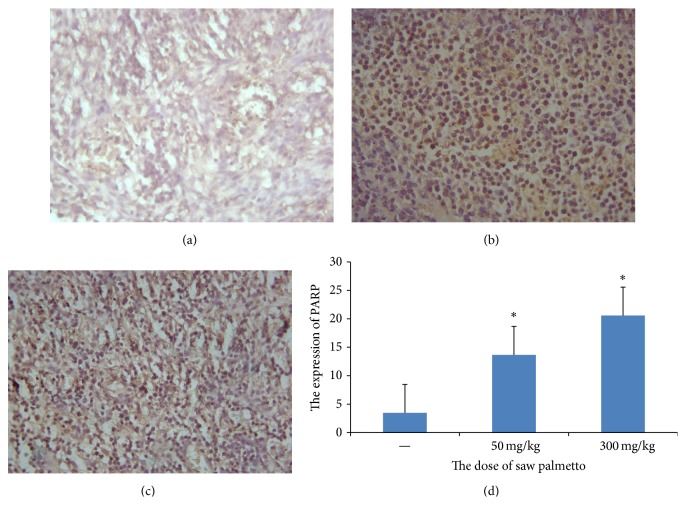
The expression of PARP in the glioma tissue. (a) is the control group, (b) is the low dose group in which the rats received administration of 50 mg/kg saw palmetto extract every day for 4 weeks, and (c) is the high dose group in which the rats received administration of 300 mg/kg saw palmetto extract every day for 4 weeks. (d) is the histogram to evaluate the expression of PARP in the three groups. *∗* means *p* < 0.05.

**Figure 6:**
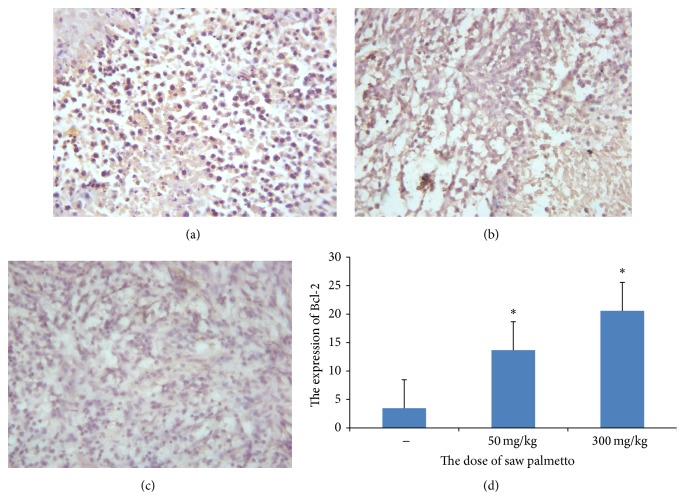
The expression of Bcl-2 in the glioma tissue. (a) is the control group, (b) is the low dose group in which the rats received administration of 50 mg/kg saw palmetto extract every day for 4 weeks, and (c) is the high dose group in which the rats received administration of 300 mg/kg saw palmetto extract every day for 4 weeks. (d) is the histogram to evaluate the expression of Bcl-2 in the three groups. *∗* means *p* < 0.05.

Furthermore, the proapoptotic effect of saw palmetto was explored by immunohistochemistry assay. The Bcl-2 and PARP (poly ADP-ribose polymerase) were detected in this study. In the study of cell apoptosis, PARP as the DNA repair enzyme which can repair the DNA is a core member of apoptosis. It plays an important role in DNA damage repair and apoptosis. PARP can be used as the hallmark of apoptosis. The average optical density is 0.313 ± 0.137 in control group, 0.238 ± 0.127 in low dose group, and 0.119 ± 0.078 in the high dose group (*p* < 0.05) (see Figure 5). Bcl-2 is an antiapoptosis protein. In this study we also detect the expression of Bcl-2 to further certify the effect of saw palmetto on the glioma cell. The average optical density is 0.116 ± 0.010 in control group, 0.285 ± 0.014 in low dose group, and 0.335 ± 0.016 in the high dose group (*p* < 0.05) (see Figure 6).

## 4. Discussion

Human gliomas are tumors of glial origin of central nervous system (CNS) and they are the most common primary tumors, accounting for ~46% of intracranial tumors and ~2% of adult tumors. The glioma is characterized by its invasive growth and is difficult to be treated via surgery. In addition, the human glioma cell is not sensitive to chemotherapy and radiotherapy.

In previous years, studies have focused on chemical compounds which derived from plants that possess pharmacological activity in antitumor therapy [10, 11]. A previous study demonstrated that a number of chemical compounds in herbaceous plant sources can inhibit the proliferation of tumor cells and induce apoptosis through altering the tumor metabolism [12].

The effective ingredients of saw palmetto extract, as matured dry fruit of saw palm, are fatty acids. A foreign study reported that saw palmetto extract could inhibit the proliferation and induction of apoptosis in prostate cancer cells [13]. At the same time, other studies have found that saw palmetto extract can effectively inhibit multiple myeloma, breast cancer, and another tumor cell proliferation. In this study we detected cell apoptosis by TUNEL method. The number of positive cells in the experimental group was significantly higher than that in the control group. In addition, the apoptosis ratio is higher in the high dose group than the low dose group. The results suggest that saw palmetto extract can increase the apoptosis of glioma cells. At the same time, the presence of some apoptotic related proteins was detected in U87 and U251 glioma cells.* Serenoa repens* significantly increased the level of both cleaved PARP. These results suggest that saw palmetto extract is a kind of antiglioma effect of drugs.

Glioma is one of the most common malignant tumors with the highest degree of vessels in intracranial tumor. It is typical angiogenesis dependent tumor which makes its invasion and recurrence rate significantly higher than those of other intracranial tumors [14–16]. The study found that the brain glioma is involved in vascular endothelial cell activation, proliferation, migration, basement membrane and extracellular matrix degradation, endothelial cell remodeling, and cell interactions with surroundings. In human glioma angiogenesis, proliferation of endothelial cells must break through the barrier of the extracellular basement membrane. The degradation of matrix in tumor angiogenesis is primarily completed by the metal matrix protease (MMPs) secreted by tumor cells. Many studies have shown that MMPs are directly associated with tumor angiogenesis, tumor invasion, and metastasis of malignant tumor [17, 18]. MMPs can lead to the formation of new blood vessels and enhance the invasion and metastasis ability of tumor. Hamasuna et al. [19] confirmed that MMP-2 can be used as a new index of tumor malignant degree and prognosis. Recent studies have also confirmed that the synthesis and activation of MMP-2 can be induced by glioma. The activation of MMP-2 not only promotes the formation of new blood vessels in the host tissue but also maintains the integrity of the vascular structure. The volume of glioma model cultured by C6 cell line in rats can be significantly reduced, treated by MMP-2 specific inhibitor factor (TIMP-2), and accompanied with the degeneration and necrosis of blood vessel.

Study on the mechanism of glioma angiogenesis and antiangiogenesis has become a new method for the treatment of glioma in recent years. In this study we adopted immunohistochemistry assay to detect CD34, MMP-2, and other indicators in glioma tissue. The results suggested that CD34 optical density value of the experimental group was higher than that of control group. As is known, CD34 is a good marker to reflect the density of blood vessels in the tumor. CD34 is closely related to tumor occurrence, development, and prognosis [20]. This study suggests that saw palmetto extract can effectively reduce the expression of CD34 in tumor cells, so that it can effectively inhibit the tumor angiogenesis. At the same time, we detected the expression of MMP-2 and the results revealed that MMP-2 in the control group was also significantly higher than that in the experimental group. According to the above results it is suggested that saw palmetto extract reduces tumor angiogenesis rough reducing the degradation of extracellular matrix by inhibiting expression of MMP-2 protein.

Signal transducer and activator of transcription factor 3 (STAT3) is a bifunctional protein coupled with tyrosine phosphorylation signal pathway, which exists in the cell holder and regulates the expression of a variety of related functional protein, cell proliferation, and apoptosis [21–23]. STAT3 rarely expresses in normal tissues of the human body and cells, which maintains the normal physiological function of cells and tissues. But the activation of STAT3 is persistent in the tumor tissues and cells. The study finds that STAT3 activation plays an important role in the tumor cell survival, proliferation, angiogenesis, invasion, metastasis, and immune escape [24]. There is high expression of STAT3 in many kinds of tumor cells, so the research of STAT3 in tumors has become a hot topic. The present study has proven that there is a close relationship between STAT3 and cell adhesion molecules, extracellular matrix degrading enzymes, angiogenesis, metastasis, and promotion tumor angiogenesis through MMP [25]. The activation of oncogene STAT3 expression can be induced by antiapoptotic proteins such as Bcl-2, MD-1, and Bcl-XL. All of these are known to promote tumor growth.

The tyrosine of STAT3 is activated under the combination cytokines, growth factors, and hormones with its receptor. STAT3 combined with Janus kinase (JAK) in the cytoplasm caused its tyrosine and JAK tyrosine phosphorylation. The STAT3 is activated after the tyrosine phosphorylation. After STAT3 activated, STAT3 forms homo- or heterodimer in cytoplasm and quickly enters the nucleus to combine with the specific gene promoter on the transcription of target genes, such as the antiapoptotic genes Bcl-2 and Mcl21, cell cycle control genes c-myc and cyclinDl, and angiogenesis related genes VEGF. Studies show that STAT3 phosphorylation at tyrosine 705 is abnormal aggregation in malignant glioma cells, particularly in glioblastoma. The expression of pY705-STAT3 is positively correlated with tumor grade and is one of the poor prognosis factors in survival analysis [26]. The results show that the abnormal activation of STAT3 plays an important role in the occurrence and development of malignant glioma. Rahaman et al. [27] reported that inhibiting the activity of STAT3 could reduce the proliferation of glioma cells and even promote the apoptosis of glioma cells. Meanwhile inhibition of the activation of STAT3 can decrease the expression of Bcl-2 and Bcl-XL. Abnormal p-STAT3 aggregation can also be observed in the proliferation of vascular endothelial cells which means that p-STAT3 is involved in tumor angiogenesis. In malignant glioma, the expression of activated STAT3 can increase the expression of vascular endothelial growth factor (VEGF). The target genes that have been identified in the STAT3 coding are antiapoptotic proteins Bcl-2 and Bcl-XL, proliferation related proteins Cyclin Dl and Myc, and angiogenesis factor VEGF.

As mentioned, matrix metalloproteinases (MMPs) are a key enzyme in the degradation of extracellular matrix. The study found that STAT3 can bind to MMP-2 promoter to enhance the expression of MMP-2 [28]. Tumor development depends on angiogenesis in order to get the nutrients required for growth and metastasis. It is known that many growth factors and cytokines are involved in the regulation of tumor angiogenesis. Vascular endothelial growth factor plays an important role in the tumor angiogenesis [29]. STAT3 is involved in the regulation of transcription of VEGF. Study has confirmed that the VEGF promoter has the binding site with STAT3. STAT3 can directly bind to the promoter of VEGF further to upregulate the expression of VEGF in tumor cells.

In summary, we found that saw palmetto extract was an important phytotherapeutic drug against the human glioma through STAT3 signal pathway. Saw palmetto extract has been widely used to treat benign prostatic hyperplasia and androgenic alopecia in clinic; the related adverse reactions included ejaculatory disorders, postural hypotension, dizziness, headache, gastrointestinal disorders, rhinitis, fatigue, and asthenia, but the side-effects were limited [30, 31] except for children [32]. So, saw palmetto extract may be useful as an adjunctive therapeutic agent for treatment of individuals with glioma and other types of cancer in which STAT3 signaling is activated.
